# Quantifying Biomass Changes of Single CD8+ T Cells during Antigen Specific Cytotoxicity

**DOI:** 10.1371/journal.pone.0068916

**Published:** 2013-07-23

**Authors:** Thomas A. Zangle, Daina Burnes, Colleen Mathis, Owen N. Witte, Michael A. Teitell

**Affiliations:** 1 Department of Pathology and Laboratory Medicine, University of California Los Angeles, Los Angeles, California, United States of America; 2 Department of Molecular and Medical Pharmacology, University of California Los Angeles, Los Angeles, California, United States of America; 3 Department of Microbiology, Immunology, and Molecular Genetics, University of California Los Angeles, Los Angeles, California, United States of America; 4 Eli and Edythe Broad Center of Regenerative Medicine and Stem Cell Research, University of California Los Angeles, Los Angeles, California, United States of America; 5 Howard Hughes Medical Institute, University of California Los Angeles, Los Angeles, California, United States of America; 6 Bioengineering Interdepartmental Program, University of California Los Angeles, Los Angeles, California, United States of America; 7 Molecular Biology Institute, Jonsson Comprehensive Cancer Center, and California NanoSystems Institute, University of California Los Angeles, Los Angeles, California, United States of America; University of Cape Town, South Africa

## Abstract

Existing approaches that quantify cytotoxic T cell responses rely on bulk or surrogate measurements which impede the direct identification of single activated T cells of interest. Single cell microscopy or flow cytometry methodologies typically rely on fluorescent labeling, which limits applicability to primary cells such as human derived T lymphocytes. Here, we introduce a quantitative method to track single T lymphocyte mediated cytotoxic events within a mixed population of cells using live cell interferometry (LCI), a label-free microscopy technique that maintains cell viability. LCI quantifies the mass distribution within individual cells by measuring the phase shift caused by the interaction of light with intracellular biomass. Using LCI, we imaged cytotoxic T cells killing cognate target cells. In addition to a characteristic target cell mass decrease of 20–60% over 1–4 h following attack by a T cell, there was a significant 4-fold increase in T cell mass accumulation rate at the start of the cytotoxic event and a 2–3 fold increase in T cell mass relative to the mass of unresponsive T cells. Direct, label-free measurement of CD8+ T and target cell mass changes provides a kinetic, quantitative assessment of T cell activation and a relatively rapid approach to identify specific, activated patient-derived T cells for applications in cancer immunotherapy.

## Introduction

CD8+ T lymphocyte mediated cytotoxicity is a critical component of the adaptive immune response against viruses and cancers, and is also implicated in autoimmunity [1], [2]. T cell mediated cytotoxicity is typically measured by target cell death or surrogate markers of effector cell cytotoxic capacity. The canonical assays are the ^51^Cr release assay and ELISPOT, both of which provide bulk measurements of whole lymphocyte population or sub-population responses [3], [4]. The introduction of peptide-MHC tetramers and microfluidic platforms has allowed for surrogate measures of cytotoxicity through analysis of T cell antigen specificity and cytokine secretion [3], [5], [6]. Directly tracking T lymphocyte mediated cytotoxicity at the single cell level is advantageous for analyzing cytotoxic T cells (CTLs) within a mixed population, which is of particular relevance in assessing T cell recognition against cancer cells. Viable CTLs can potentially be cultured and expanded further, or the corresponding T cell receptors (TCRs) bearing optimal specificity toward immunogenic peptides can be molecularly cloned for utilization in a clinical setting [7].

Optical microscopy allows for direct identification and tracking of CTLs in the full context of target cell recognition and killing. Optical imaging methods such as epifluorescence, confocal microscopy, total internal reflection fluorescence and two photon laser scanning microscopy have been explored for the study of lymphocyte activation, but typically require antibody or conjugated protein labeling to track and quantify cells [8], [9]. This limits applicability to studies of T lymphocytes due to transduction inefficiencies associated with diverse phenotypes as well as progressive differentiation towards exhaustion or senescence during in vitro culture, as is required for typical fluorescence labeling techniques [10], [11]. Live cell interferometry (LCI) is a label-free optical microscopy technique which measures whole cell responses. LCI uses a Michelson-type interferometer to compare the optical thickness of living cells in a sample chamber to the optical thickness of fluid in a reference chamber in order to quantify the optical thickness difference between a cell and its surrounding media [12], [13]. The optical thickness difference due to the interaction of light with cellular biomass is linearly proportional to the material density of a cell [14]. Based on this interaction, cell mass can be related to the measured phase retardation of light passing through each cell with 2% precision in total cell mass [12]–[14]. Practically, LCI yields measurements of mass and mass accumulation or loss rates of 100–400 cells simultaneously per imaging location within 1–5 h of imaging [12]. With automated measurements every 2–5 minutes to allow for accurate tracking and mass determination during cytotoxic events at 20–50 imaging locations, this technique can quantify the mass of 2,000 to 20,000 cells.

Our approach directly tracks T lymphocyte mediated cytotoxicity at the single cell level without labeling by quantifying the mass of individual CTLs and their cognate target cells. Single cytotoxic events are identified and evaluated over time within a mixed population, using the mass data to confirm individual T cell mediated cytotoxicity events. As a proof of concept, we demonstrate tracking of up to 2,000 individual CTLs with specificity toward Melanocytic Antigen Recognized by T lymphocytes (MART1) responding against human leukocyte antigen (HLA) matched MART1+ target cells [15]. Target cells are imaged by the LCI to establish a base-line mass accumulation rate. CTLs are then plated onto the target cells and individual cytotoxic events are identified as a characteristic decrease in target cell mass following contact with a corresponding T cell.

It is well established that T cells increase in size during activation [16]. This previously observed increase in size may result from a change in solute concentration or osmolality within the cell as opposed to an increase in biomass [17]. Using our approach we determine that the size increase in CTLs responding to cognate target cells is due to an increase in biomass and that biomass measurements provide robust identification of activated T cells. The capacity to measure the mass of a single CTL opens several potential downstream applications including T cell biological studies pertaining to metabolic or differentiation states in addition to the analysis of CTLs for potential use in adoptive immunotherapy protocols.

## Materials and Methods

### Cell Lines & PBMCs

M202, M207, [18] PC-3, PG13, and 293T cells (ATCC) were routinely maintained at 37°C in 8% CO_2_, using either DMEM or RPMI1640 Media supplemented with 5% FBS, 100 U/mL penicillin, 100 µg/mL streptomycin and 2 mmol/l-glutamine. HLA A2.1+ PBMCs derived from anonymized healthy donors were obtained from the Center for AIDS Research Virology Core Lab at UCLA and frozen following collection. Thawed PBMCs were revived in complete medium (CM) plus anti-CD3/2/28 beads for 4 d prior to retroviral infection. CM consisted of AIM-V media (Invitrogen, USA) supplemented with 25 mmol/L HEPES, 5.5×10^−5^ mol/L β-mercaptoethanol and 300 IU/mL IL-2. PBMCs were in culture for a total of 7–10 d prior to all imaging experiments. Cells were maintained in complete media on the LCI imaging platform.

### Generation of MART1 specific CD8+ T cells

F5 retrovirus was collected from PG13 cells modified to produce retroviral vector consisting of the F5 TCR with specificity toward the MART1 ELAGIGLTV peptide fragment, which is expressed by the M202 and M207 cell lines used in cytotoxicity experiments. Briefly, 293T cells were transfected with the packaging vector pCL-Eco and the MSCV-based retroviral vector RV-MSCV-F5MART1 TCR. Resulting supernatants were used to transduce the murine PG13 retrovirus packaging cell line for Gibbon ape leukemia virus (GaLV) envelope-pseudotype generation. PBMCs were infected with the retrovirus containing PG13 supernatant in the presence of Retronectin (Takara, Japan) according to the manufacturer's protocol. 48–72 h after infection the cells were stained with MART1 specific tetramer (Beckman Coulter, USA) and analyzed by flow cytometry (FACSCanto, BD Biosciences, USA). CD8+ T cells were isolated by negative enrichment (Stem Cell Technologies, USA) and the enrichment efficiency was verified by flow cytometry.

### IFNg measurement by flow cytometry

To verify the functional specificity of DMF5 transduced CD8+ T cells, a total of 1×10^5^ T cells were co-cultured with 1×10^5^ target cells (M202 or M207) in a 96-well flat plate with 200 µl of complete medium in a humidified incubator at 37°C and 8% CO_2_ for 18 h. The concentration of IFN-gamma in the supernatant was determined by flow cytometry using the Human IFNg FlowCytomix Simplex kit following the manufacturer's protocol (eBioscience, USA cat# BMS8228FF).

### LCI mass measurements

Target cells were plated onto 20 mm×20 mm silicon slides treated with a 0.01% solution of poly-l-lysine (Sigma) at a density of approximately 2.5×10^4^ cells/cm^2^ and allowed to grow in a cell culture incubator for 48 h prior to the start of imaging experiments. A silicon slide with attached target cells was placed into a custom-built, temperature and CO_2_ controlled perfusion-based live cell imaging chamber and imaged for approximately 1.5 h before the addition of T cells. The T cell-target cell co-culture was imaged continuously for 18 h. 30 imaging locations were chosen based on suitable density of target cells on the silicon substrate and images collected approximately once every 3 to 4 min. Imaging was performed using a modified GT-X8 optical profiler (Bruker) at 20× magnification (numerical aperture 0.28) with a 0.55x demagnifying lens to increase field of view while preserving resolution. Interference fringes were generated using a Michelson-type interferometer consisting of a beam splitter, reference mirror and a reference fluid chamber which compensates for the optical path length through the sample chamber. Images were acquired using the phase-shifting interferometry (PSI) method with illumination from a 530 nm fiber-coupled LED (Thorlabs). Intensity images represent the average intensity of the image without the interference fringes necessary for Michelson phase imaging.

### Phase Unwrapping

To remove the integer-wavelength phase ambiguities inherent in quantitative phase imaging [19], we performed phase unwrapping using a custom script implemented in Matlab (Mathworks). First, we performed unwrapping based on Flynn's minimum discontinuity method [19]. Next, a training dataset was constructed by manually applying single wavelength corrections to approximately 200 sub-images of the phase data, selected for the appearance of target and T cells of interest. This training dataset was used in a linear discriminant analysis (LDA) to identify pixels which lie on the boundary of phase-wrapped regions, based on 16 sets of image statistics, including the raw image itself, the computed intensity image, and the results of various edge-finding filters applied to the wrapped phase image. LDA was followed by genetic optimization to refine the LDA results and watershed algorithm thresholds used in determining the boundaries of phase-wrapped regions. Regions within the boundaries determined by the watershed algorithm applied to the final LDA result were shifted (corrected) by a phase shift of one wavelength and median filtered with a kernel size of 3.

### Mass Tracking

Single cell mass measurements were performed using a custom script implemented in Matlab (Mathworks). Briefly, phase-corrected images were Gaussian low pass-filtered before image segmentation based on Otsu thresholding. Finally, objects identified by image segmentation were tracked using the particle tracking code adapted for Matlab by Daniel Blair and Eric Dufresne, based on the particle tracking algorithm by Grier *et al.* [20]. Cell area was determined using a local adaptive threshold based on a 200 pixel neighborhood in the quantitative phase image.

### Statistics

Statistical analysis was performed using a two-tailed Welch's Student T test with unequal variances and sample sizes.

## Results

### LCI for quantitative imaging of T cell mediated cytotoxicity

**Figure 1:**
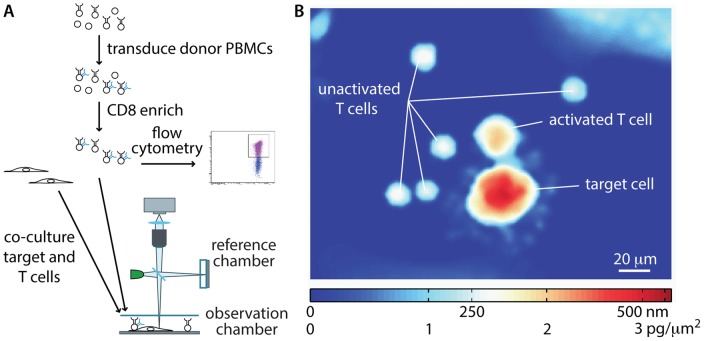
LCI measures mass of T and target cells. (A) Workflow for T cell mass measurement experiments. Donor peripheral blood mononuclear cells (PBMCs) are transduced with the MART1 specific, F5 TCR and enriched for CD8+ T cells. A subset of these T cells are analyzed by flow cytometry to confirm a transduction efficiency of greater than 50%. The remaining cells are imaged on the LCI with MART1 expressing, HLA-matched (or mismatched control) M202 target cells. (B) Sample LCI data showing the phase shift and mass distributions in an activated, F5-transduced CD8+ T cell, several unresponsive T cells, and a dying target cell.



We developed a model system for analyzing cytotoxicity events by establishing the antigen specificity of healthy human donor CD8+ enriched lymphocytes against HLA matched target cell lines. Peripheral blood mononuclear cells (PBMCs) were transduced with an F5 anti-MART1 TCR, which is a high affinity TCR with specificity toward MART1 [15]. Target cells expressing MART1 and antigen-defined CD8+ enriched T cells were co-cultured in a live-cell observation chamber on the LCI stage and imaged for a period of 18 h. (Figure 1A). The observation chamber was temperature controlled with pH maintained by continuous perfusion of media equilibrated at 8% CO_2_. Following image collection, the light phase shift data was corrected for phase wrapping errors which are caused by the integer wavelength ambiguity inherent in quantitative phase imaging [19]. The result is a map of phase shifts across each cell that can be converted into a map of local dry mass density (Figure 1B). The total dry mass of a cell is quantified as the sum of the local densities [12], [14], [21]:

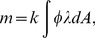
(1)where m is cell dry mass, φ*λ* is the measured phase shift, *k* is the mass conversion factor, and *A* is projected area. The mass conversion factor [21], [22], which is a measure of the change in density per unit change in refractive index (Δ*ρ*/Δ*n*), is taken as *k* = 5.56 pg/μm^3^ [14]. This parameter, *k*, is measured as a change in refractive index relative to the refractive index of water, Therefore, the cell mass measured in this manner is the cell dry mass, or the mass of everything within the cell other than water. With this equation, the measured dry mass of the activated T cell in Figure 1B is 240 pg, the target cell mass is 840 pg and the unactivated T cells have an average dry mass of 65 pg.

### Antigen-specific T cells and maintenance of viability on the imaging platform

**Figure 2:**
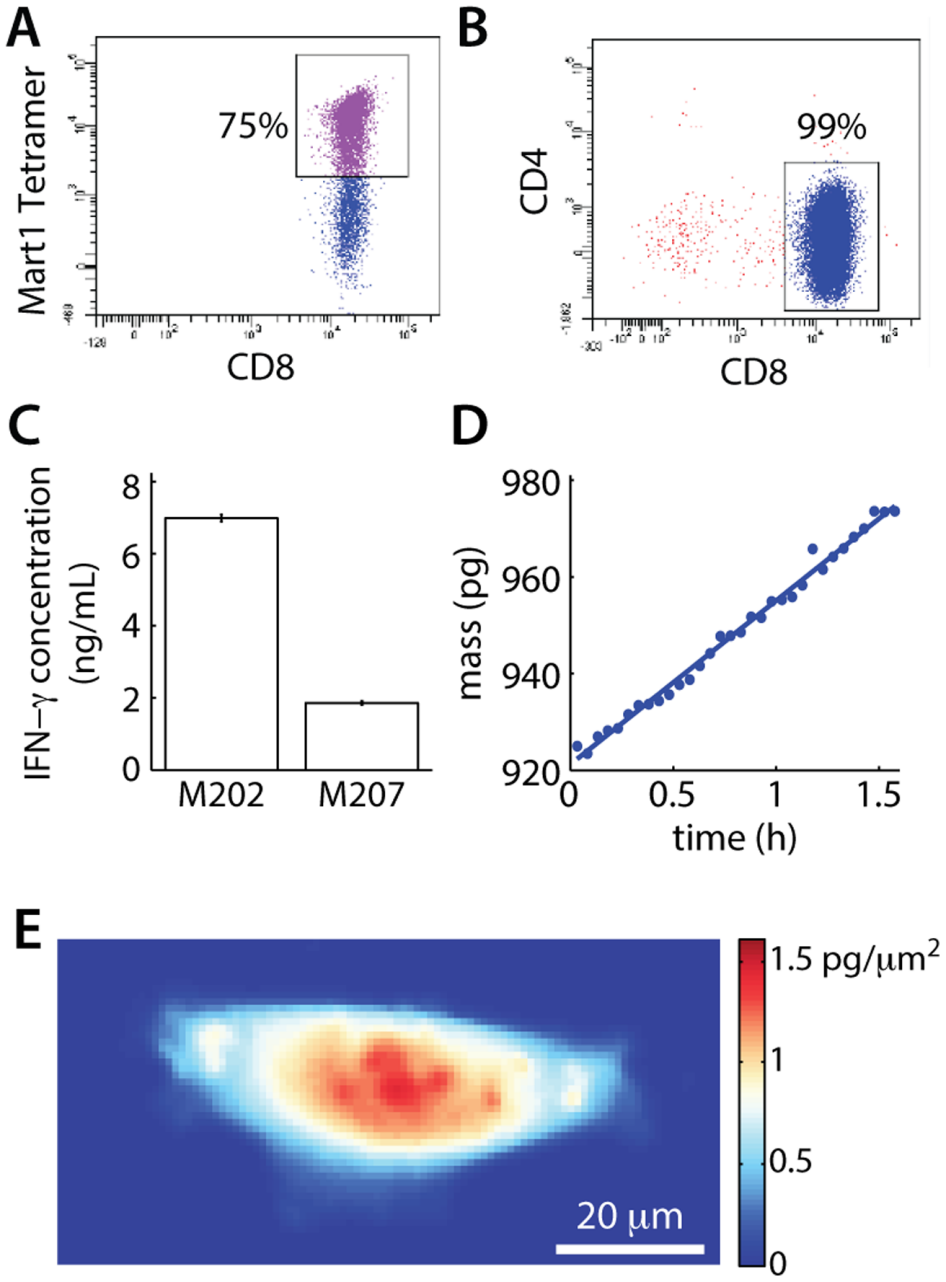
Transduction of CD8+ enriched PBMCs. (A) Flow cytometry data of transduced T cells showing typical transduction efficiency of donor PBMCs. (B) Flow cytometry of CD8+ enriched population showing CD8+ T-cell enrichment efficiency. (C) IFNg release assay validating F5-transduced, CD8+ enriched T cell activation following co-culture with HLA-matched MART1 expressing M202 cells. Negative control M207 cells express MART1, but are HLA-mismatched. (D) Mass vs. time of the healthy M202 cell shown in (E), demonstrating the viability of target cells on the interferometer stage.

To generate antigen-defined CTLs, we infected HLA A2.1+ healthy donor PBMCs with the F5 TCR by retroviral transduction and enriched for CD8+ cells by magnetic separation to remove magnetically labeled non-CD8+ cells (Figure 2A–B). Although CD8+ T cells have endogenous TCRs, ectopic expression of the F5 anti-MART1 TCR results in overexpression of the exogenous alpha and beta chains to allow for preferential pairing and surface expression. The majority of isolated cells were CD8+ with 75% expressing the F5 TCR on the surface, as determined by MART1 peptide tetramer stains prior to imaging. We measured interferon gamma (IFNg) accumulation in the supernatant following an 18 h co-culture period to verify that F5 redirected CD8+ T cells were specific for the cognate target cells. Results of a bead-based immunoassay analyzed by flow cytometry indicated a significant, 3.5-fold higher, IFNg release from F5 transduced CTLs upon co-culture with HLA-matched MART1+ M202 target cells as compared to co-culture with an HLA-mismatched control cell line (Figure 2C).

Target cells were imaged in standard culture media for 1.5 h prior to the start of each experiment to confirm the live cell culture imaging platform maintains viability of target cells in the absence of CTLs. M202 target cells showed a positive mass accumulation rate, indicating a healthy population and the maintenance of cell viability. (Figure 2D–E and Figure S1B). Control experiments demonstrated maintenance of both T and target cell viability during extended imaging periods (Figure S1 and Figure S2).

### Mass decrease of killed target cells

**Figure 3:**
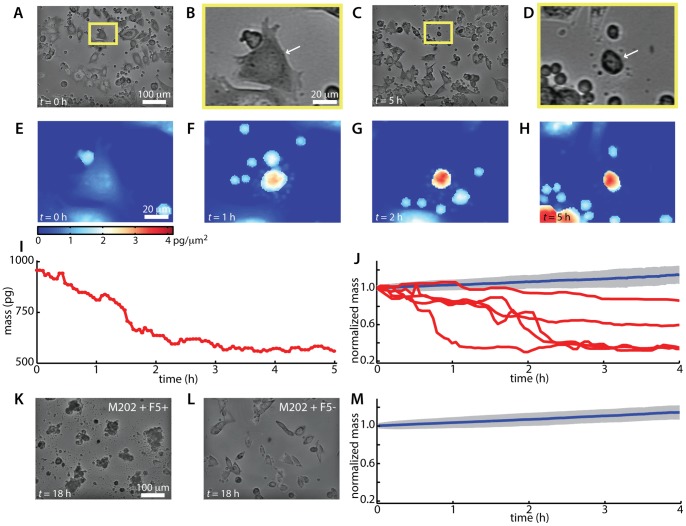
LCI tracks target cell death during T cell mediated cytotoxicity. (A–H) Images of a single cytotoxic event occurring immediately after the start of imaging (*t* = 0 is approximately 30 min after plating CTLs onto target cells), (A–D) intensity images at *t* = 0 and 5 h of imaging demonstrating CTL mediated target cell killing. Yellow boxes in (A) and (C), indicate the subregion in images (B) and (D). Arrows in (B) and (D) indicate the target cell tracked by mass profiling in (E–I). (E) LCI mass profile of selected target cell after initiation of persistent contact with a target cell at the start of imaging. (F–H) LCI mass profile of dying target cell. (I) Measured total mass vs. time for target cell shown in (E–H). (J) Normalized mass of killed and healthy target cells over time. Normalized mass is mass divided by initial mass. Healthy cells show roughly 15% increase in normalized mass over 4 h (blue line indicates mean of *n* = 311 healthy M202 cells, grey region indicates +/− SD). Killed target cells (red lines) show a decrease in mass of 20 to 60% over 1–4 h. (K) intensity image of stage location shown in (A) and (C) after 18 h of imaging, showing nearly complete death of target cells. (L) Intensity image of stage after 18 h of imaging M202 cells plated with untransduced (F5-) CD8+ T cells showing viability of target cells plated with nonspecific T cells. (M) Normalized mass vs. time for *n* = 2058 healthy M202 cells treated with untransduced, control CTLs, showing roughly 15% increase in mass over 4 h.

After 1.5 h of target cell control measurements, F5 MART1 reactive CTLs (Figure 2A–B) were added to the live cell imaging chamber and imaged continuously for 18 h. This experiment duration is similar to the time period typically required for measurement of T cell activity by ELISPOT [3]. Single CTLs killing individual target cells are identified through qualitative analysis of the intensity image data as a change in appearance of the target cell following prolonged contact with a CTL (Figure 3A–D). Cytotoxic events are detectable despite the presence of nonspecific or unresponsive T cells within the broader population. LCI provides quantitative maps of the mass distribution within target cells during T cell mediated cytotoxic events (Figure 3E–H). These mass distributions from successive image frames can be integrated to yield measurements of target cell mass over time (Equation 1 and Figure 3I). Individual cytotoxic events due to recognition of CTLs are confirmed by a characteristic decrease in target cell mass following prolonged contact (30 min to 2 h) with a corresponding CTL (Figure 3I and Movie S1).

Target cell mass decreased by 20 to 60% over a period of 1–4 h when successfully attacked by a CTL, as compared to an increase in total target cell mass of 15% over 4 h when not killed by CTLs (Figure 3I–J). Despite contact between T cells and target cells, there was no response in control experiments using HLA mismatched, antigen irrelevant target cells (lacking MART1) or non-specific T cells (Figure 3 K–M, Figure S1C–D and Figure S3C–D). This indicates that target cell death was due to the presence of antigen-specific CTLs and that the rate and extent of target cell mass decrease due to T cell mediated cytotoxicity is directly quantifiable using LCI. T cell mediated cytotoxicity is evident within the first 30 min and confirmed within the first 2–4 h following the addition of CTLs, indicating the speed of the LCI approach in measuring T cell mediated cytotoxicity (Movie S1). An estimated 95% of target cells were dead by 18 h after the addition of CTLs, while greater than 95% of control target cells appeared healthy at 18 h (Figure 3 K–L and Figure S3).

### Mass increase of activated CTLs

**Figure 4:**
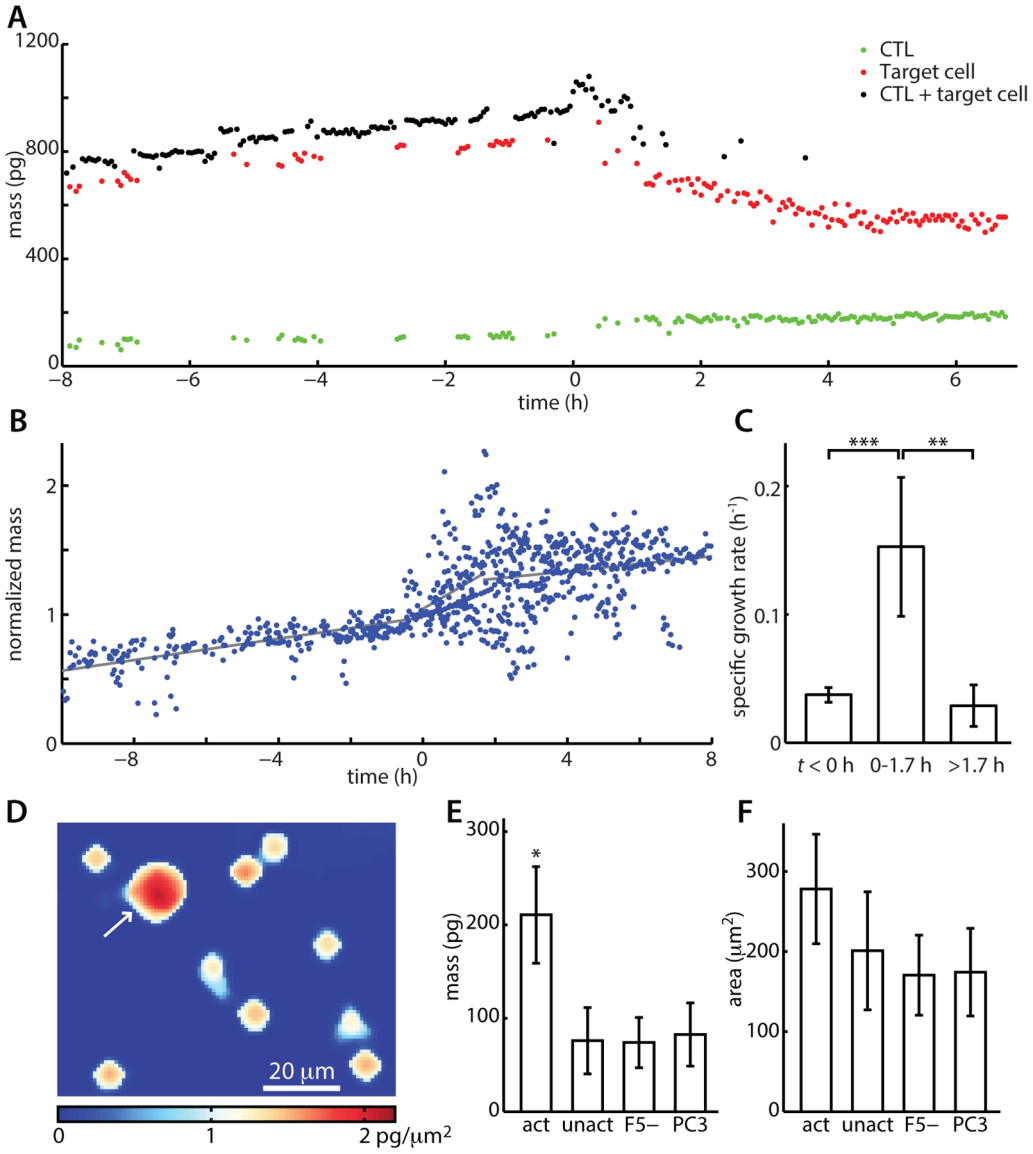
LCI measures CTL mass and mass accumulation rate during T cell mediated cytotoxicity. (A). Mass versus time of an activated CTL and corresponding target cell. *t* = 0 h is the point at which the target cell detaches from the substrate at the beginning of cell death. CTL + target cell refers to total mass of both cells in frames where they could not be measured separately. (B) Normalized mass versus time of 10 CTL-mediated cytotoxicity events. CTL mass is normalized relative to the mass at the time of target cell morphology change, which is used as the *t* = 0 h point for all traces. Gray lines show best fit lines used for determining mass accumulation rates. (C) Average mass accumulation rate of CTLs before a cytotoxic event, during the first 100 min of a cytotoxic event, and after the first 100 min of a cytotoxic event demonstrating an approximately 4-fold increase in mass accumulation during the first 100 min of a cytotoxic event. (D) LCI image of 9 unresponsive and 1 cytotoxic T cell illustrating an approximately 3-fold difference in mass. The white arrow indicates the activated T cell, as determined by tracking this cell after persistent contact with target cell and subsequent target cell death. (E) The average mass of 116 activated CTLs is approximately 2.8-fold greater than the average mass of unresponsive controls. (F) Average area of activated CTLs is only approximately 1.4-fold greater than non-activated controls and not significant at the 95% confidence level, illustrating the utility of LCI mass measurements for determining CTL activation. Error bars in C show 95% confidence intervals. Error bars in E and F show +/− SD. * *p*<0.05, ** *p*<0.01, *** *p*<10^−3^. act  =  activated/cytotoxic, 116 cells, *n* = 3 experiments. unact  =  unactivated/unresponsive, 359 cells, *n* = 3 experiments. F5-  =  untransduced, F5-negative control experiment, 530 cells, *n* = 2 experiments. PC3  =  PC3 cell, HLA-mismatched irrelevant antigen control, 3015 cells, *n* = 3 experiments.

In parallel with the decrease in target cell mass, individual activated CTLs increased in overall size by the end of a cytotoxic event (Figure 4). Individual CTL and target cell masses can be tracked through the duration of their interactions (Figure 4A and Figure S4). CTL mass versus time data for 10 such events is summarized in Figure 4B, with CTL mass normalized relative to the mass when the target cell dramatically changed morphology (“balled-up”) at the start of a death event, which is defined as *t* = 0 h. In a typical trace, the target cell initially shows an increase in mass consistent with the growth rate of a healthy cell (Figure 3M). During this period (*t*<0 h), CTLs show a relatively slow growth rate (Figure 4C). Then, the target cell “balls-up” and detaches from the substrate, immediately prior to a very rapid loss of mass over the first 1–2 hours. During this initial period (approximately 100 min), the T cell mass accumulation rate increases significantly (Figure 4C). As the target cell loses mass and the central cell body condenses over the next 2–5 hours, the T cell continues to increase in mass, at a slower rate than during the initial period (Figure 4C).

This change in mass accumulation rate resulted in a significant 2 to 4-fold higher cellular mass than surrounding unresponsive T cells (Figure 4D). The total cellular mass of 116 CTLs at the end-point of each cytotoxic event was compared to the mass of 3,900 control T cells that did not kill targets during the course of the experiment. On average, the CTLs had a 2.8-fold higher mass as compared to their non-specific or unresponsive counterparts (Figure 4E and Figure S5A). This mass increase persisted for up to 4 h, a duration that is limited by the average period of observation prior to the activated T cell being washed away due to continuous media perfusion through the observation chamber.

The two-dimensional (2D) area of responsive versus unresponsive T cells was calculated to determine whether there was a significant difference relating to overall size. The observed 1.4-fold increase in 2D area was smaller than the 2.8-fold difference in total cell mass and did not achieve statistical significance at the *p*<0.05 level compared to controls (Figure 4F and Figure S5B). These results show that the mass change of CD8+ T cells is a more robust indicator for activity than the change in cell area. Additionally, for spherical T cells, the observed 1.4-fold increase in mass corresponds to a 1.7-fold increase in volume, which is substantially lower than the observed 2.8-fold increase in mass. These results, therefore, suggest that there is also an increase in T cell density during activation, although density quantification is not possible with the present configuration of LCI measurements.

## Discussion

LCI provides a quantitative label-free cytotoxicity assay through sensitive biomass measurements of single effector T cells and their affected target cells during cytotoxic events (Figure 1). The mass of killed target cells can be tracked over time to confirm a 20 to 60% decrease in mass over 1 to 4 h, consistent with a cytotoxic insult (Figure 3). We found a significant 4-fold increase in T cell mass accumulation rate at the start of the cytotoxic event and a 2.8-fold average increase in total mass of effector T cells after recognition and killing of cognate target cells (Figure 4). The change of mass of T cells was found to be a more significant indicator of T cell activation state than measurements of 2D changes in area alone.

The mass increase we observed in activated CTLs is likely accompanied by an increase in biosynthesis driven by metabolic changes. It has been demonstrated that T cells use glucose and glutamine as their primary energy sources. Activated lymphocytes generate energy to meet protein synthesis demands by significantly increasing glucose, amino acid and fatty acid uptake from the extracellular environment [23]. Glucose deprivation studies have shown that activated T cells require glucose for proliferation and survival even in the presence of adequate levels of glutamine [24]. TCR signaling plays a critical role in regulating the transcription of the glucose transporter *Glut1*, enabling enhanced glucose uptake with activation [25]. Studies have shown that TCR agonists such as anti-CD3 antibodies or compounds that cause cross-linking of CD3 proteins result in a rapid and maximal induction of *Glut1* expression [24], [25].

A potential application of the LCI technique presented here is for the identification and isolation of single and potentially rare CTLs. A growing body of work has focused on the identification of tumor infiltrating T lymphocytes (TILs) bearing TCR recognition of autologous tumor cells [7], [26]. Recent studies have indicated that these CTLs occur at relatively low frequencies, making it difficult to employ bulk or surrogate cytotoxicity assays to confirm their existence and isolation from a mixed population [27], [28]. The LCI approach uses the cytotoxic interaction between CTLs and target cells as a natural amplifier of the underlying peptide-MHC-TCR recognition event which avoids false positives due to nonspecific binding. The LCI imaging platform is fundamentally compatible with a segmented culture system that will allow for isolation of rare cells that may be lost in the current open perfusion cell culture system. LCI may therefore provide a viable alternative for the identification and isolation of rare effector T cells killing autologous tumor cells or HLA-matched cancer cell lines.

T cells against cancer-associated antigens are generally anticipated to bear lower affinity TCRs if they are raised against a self-antigen and presumably escaped thymic selection and tolerance induction [29]. The affinity between the TCR and peptide-MHC is considered to play a crucial role in the outcome of T cell stimulation [30]. The classic method to assess TCR-peptide-MHC affinity entails the measurement of on and off-rates using surface plasmon resonance. The surface bound peptide-MHC-TCR interaction does not accurately mimic the multiple receptor-mediated interactions that occur during recognition of a target cell by a CTL. Evidence suggests that these measurements provide limited information regarding lymphocyte effector function [30], [31]. In a transfection system, TCRs engineered with higher affinity for cognate peptide-MHC ligands compared to their wild type counterpart exhibited increased CTL activity [31]. An affinity model suggests that activation of T cells is related to the number of receptors engaged. Higher affinity interactions require less TCR-peptide-MHC engagements to activate a T cell into a cytotoxic state [32]. It is conceivable that higher affinity TCR-peptide-MHC interactions drive a more rapid response than their lower affinity counterparts, and the LCI approach may also potentially discriminate between these interactions.

## Supporting Information

Figure S1 **Averaged, normalized mass versus time plots for control target cell growth conditions showing robust growth on the LCI stage, and specificity of T cell mediated cytotoxicity.** (A) Unaffected M202 cells (*n* = 632) during treatment with F5 TCR transduced, CD8+ T cells. (B) M202 cells (*n* = 117) prior to treatment with F5 TCR transduced, CD8+ T cells. (C) M202 cells (*n* = 2058) treated with F5 TCR negative, CD8+ T cells. (D) Antigen-irrelevant, PC-3 prostate cancer cells (*n* = 1006) treated with F5 TCR transduced, CD8+ T cells. Blue line shows mean normalized mass versus time (normalized relative to mass at first timepoint). Light blue region shows the mean +/− SD.
(TIF)

Figure S2 **Averaged, normalized mass versus time for unresponsive T cells showing steady growth on the LCI stage.** (A) Unresponsive F5 TCR transduced CD8+ T cells (*n* = 101) plated with M202 target cells. (B) Untransduced CD8+ T cells (*n* = 146) plated with M202 target cells. (C) F5 TCR transduced CD8+ T cells (*n* = 950) plated with antigen-irrelevant, PC-3 prostate cancer target cells.
(TIF)

Figure S3 **Intensity images of cells on the interferometer stage after 18 h of imaging showing typical target cell conditons.** Left column shows the full image frame, the right column shows a subset of the full image frame. (A)–(D) M202 target cells plated with F5 TCR transduced, CD8+ T cells showing nearly complete death of target cells. For comparison, (A) and (B) show the same field of view as in Fig. 2 A–F. (C), (D) show a single living cell. E, F. M202 target cells plated with untransduced CD8+ T cells showing viability on the stage after 18 h of imaging and cognate TCR requirement for T cell mediated cytotoxicity. (G), (H). Antigen-irrelevant PC-3 prostate cancer target cells plated with F5 TCR transduced CD8+ T cells showing the specificity of the F5 TCR.
(TIF)

Figure S4 **(A)–(J). Mass versus time plots for CTLs and corresponding target cells, as in** **Figure 4A** **.** *t* = 0 h is the point at which the target cell detaches from the substrate at the beginning of cell death. CTL + target cell refers to total mass of both cells in frames where they could not be measured individually, typically due to overlap between the CTL and target cell.
(TIF)

Figure S5 **(A) Mass and (B) area histograms for activated and unresponsive T cells, relative to control experiments.** Activated  =  activated/cytotoxic F5 TCR transduced T cells, 116 cells, *n* = 3 experiments. Unactivated  =  unactivated/unresponsive F5 TCR transduced T cells, 359 cells, *n* = 3 experiments. F5neg  =  untransduced F5 TCR negative T cells plated with M202 target cells, 530 T cells, *n* = 2 experiments. PC3  =  F5 TCR transduced T cells plated with HLA-mismatched antigen irrelevant PC-3 prostate cancer cells, 3015 T cells, *n* = 3 experiments.
(TIF)

Movie S1 **Four panel video showing intensity images, mass distribution images, and mass vs. time of a target M202 cell being killed by a cytotoxic T cell (CD8+, F5 TCR transduced) over the course of 5 hours of observation by LCI.**
(MOV)
