# Supplementary figures and images for: Quantifying Biomass Changes of Single CD8+ T Cells during Antigen Specific Cytotoxicity
Source: PLoS One. 2013 Jul 23;8(7):e68916. doi: 10.1371/journal.pone.0068916 (PMC3720853; doi:10.1371/journal.pone.0068916)

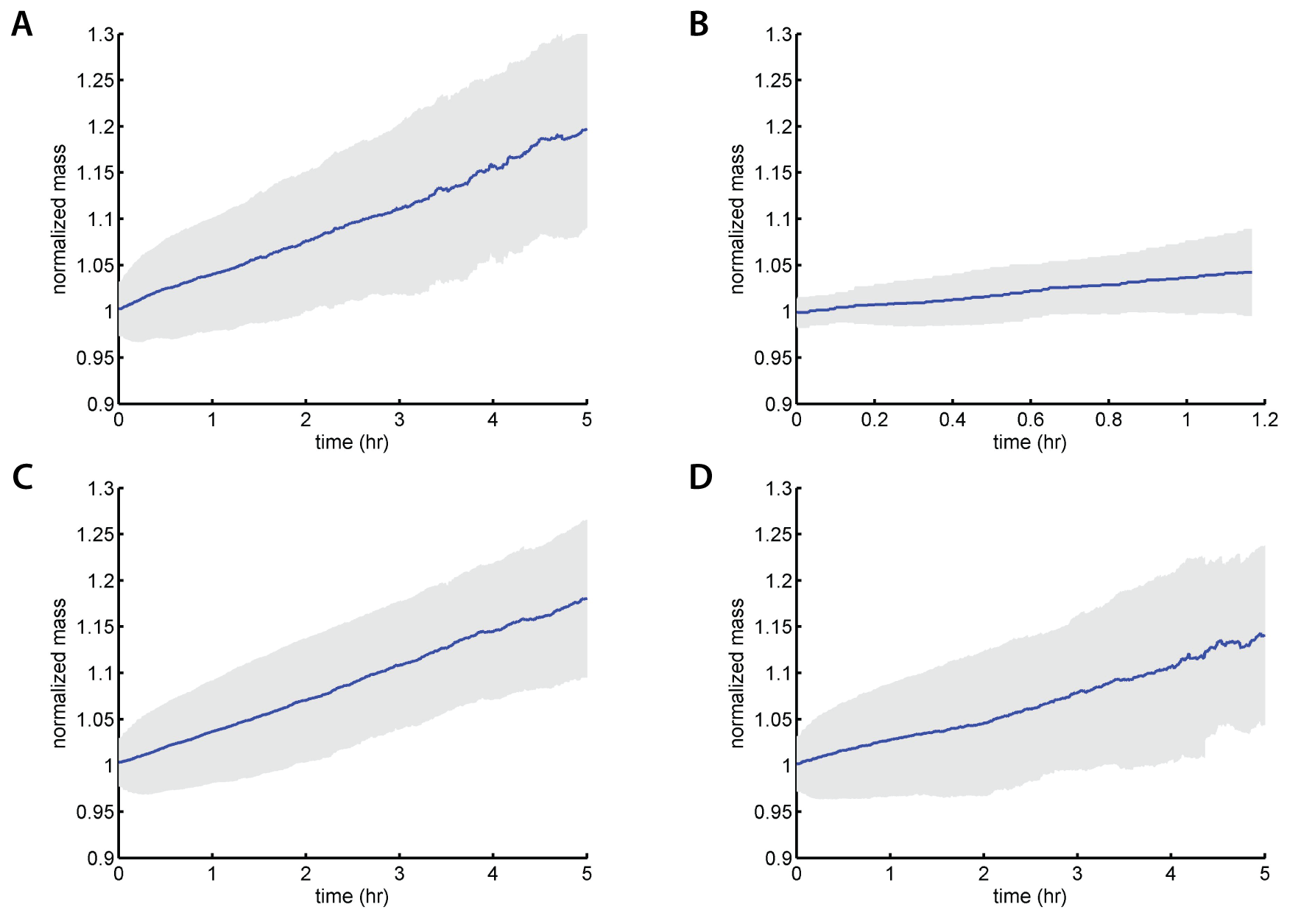

Supplement: Figure S1 — Averaged, normalized mass versus time plots for control target cell growth conditions showing robust growth on the LCI stage, and specificity of T cell mediated cytotoxicity. (A) Unaffected M202 cells (n = 632) during treatment with F5 TCR transduced, CD8+ T cells. (B) M202 cells (n = 117) prior to treatment with F5 TCR transduced, CD8+ T cells. (C) M202 cells (n = 2058) treated with F5 TCR negative, CD8+ T cells. (D) Antigen-irrelevant, PC-3 prostate cancer cells (n = 1006) treated with F5 TCR transduced, CD8+ T cells. Blue line shows mean normalized mass versus time (normalized relative to mass at first timepoint). Light blue region shows the mean +/− SD. (TIF) [file pone.0068916.s001.tif]

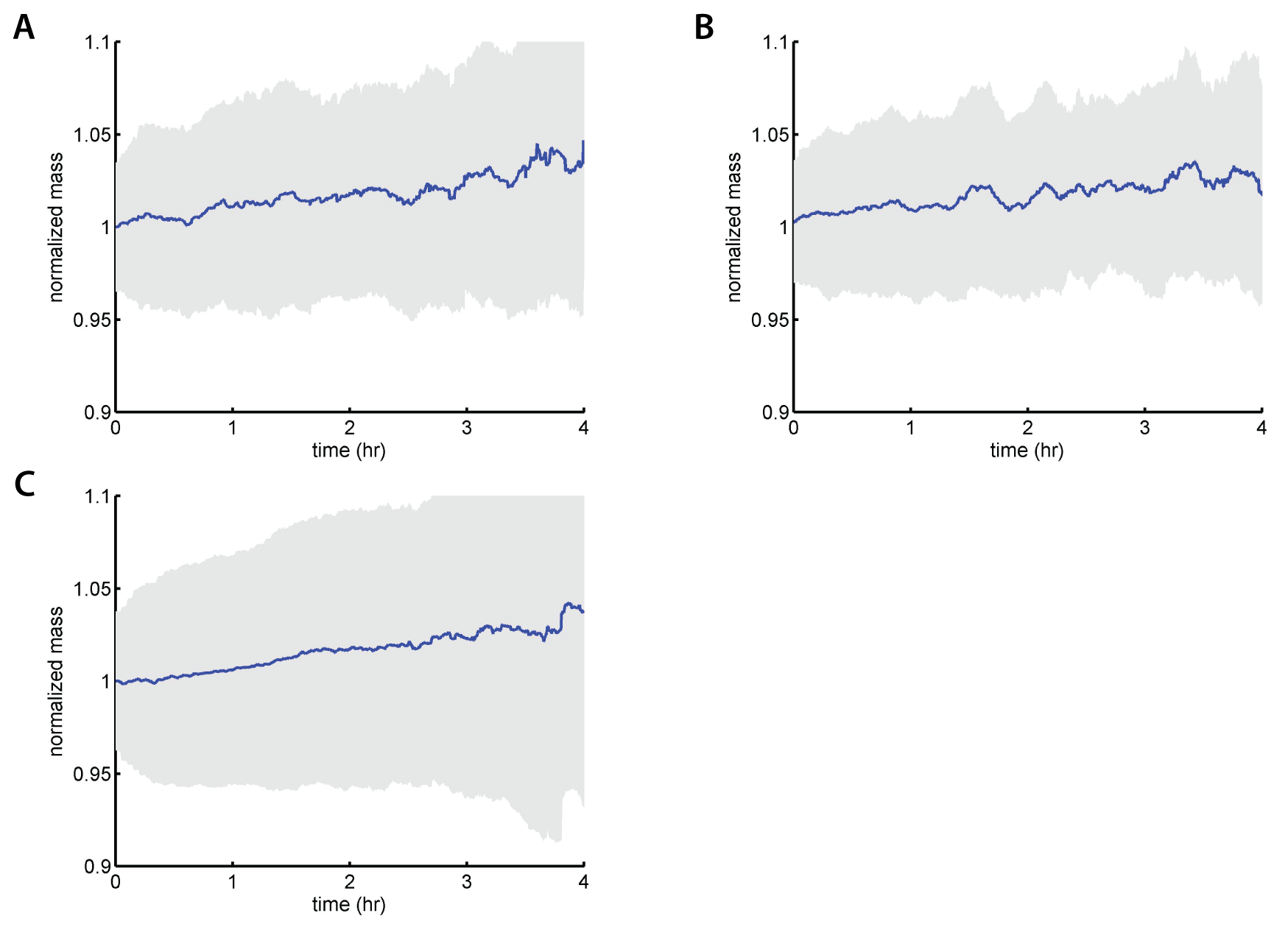

Supplement: Figure S2 — Averaged, normalized mass versus time for unresponsive T cells showing steady growth on the LCI stage. (A) Unresponsive F5 TCR transduced CD8+ T cells (n = 101) plated with M202 target cells. (B) Untransduced CD8+ T cells (n = 146) plated with M202 target cells. (C) F5 TCR transduced CD8+ T cells (n = 950) plated with antigen-irrelevant, PC-3 prostate cancer target cells. (TIF) [file pone.0068916.s002.tif]

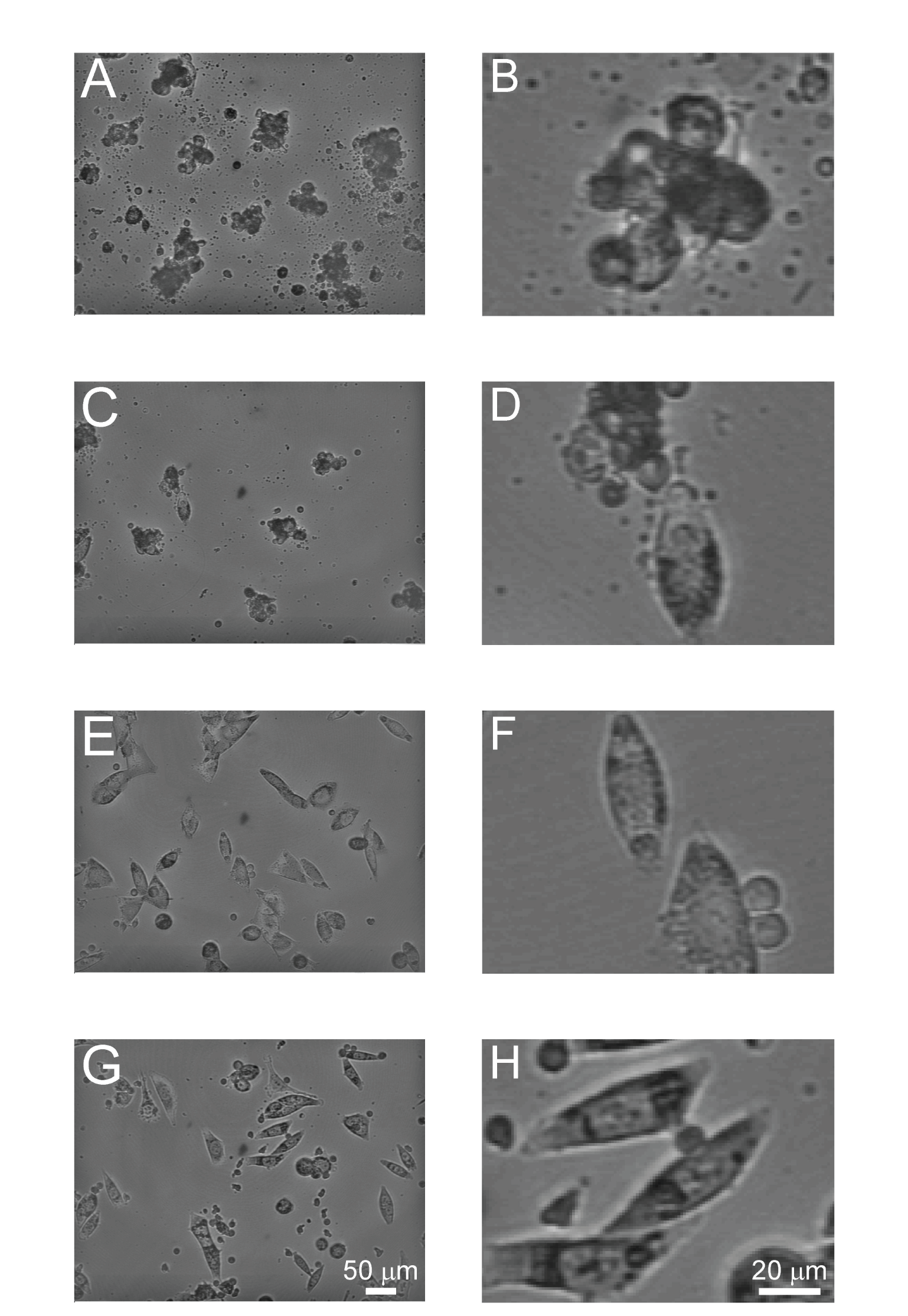

Supplement: Figure S3 — Intensity images of cells on the interferometer stage after 18 h of imaging showing typical target cell conditons. Left column shows the full image frame, the right column shows a subset of the full image frame. (A)–(D) M202 target cells plated with F5 TCR transduced, CD8+ T cells showing nearly complete death of target cells. For comparison, (A) and (B) show the same field of view as in Fig. 2 A–F. (C), (D) show a single living cell. E, F. M202 target cells plated with untransduced CD8+ T cells showing viability on the stage after 18 h of imaging and cognate TCR requirement for T cell mediated cytotoxicity. (G), (H). Antigen-irrelevant PC-3 prostate cancer target cells plated with F5 TCR transduced CD8+ T cells showing the specificity of the F5 TCR. (TIF) [file pone.0068916.s003.tif]

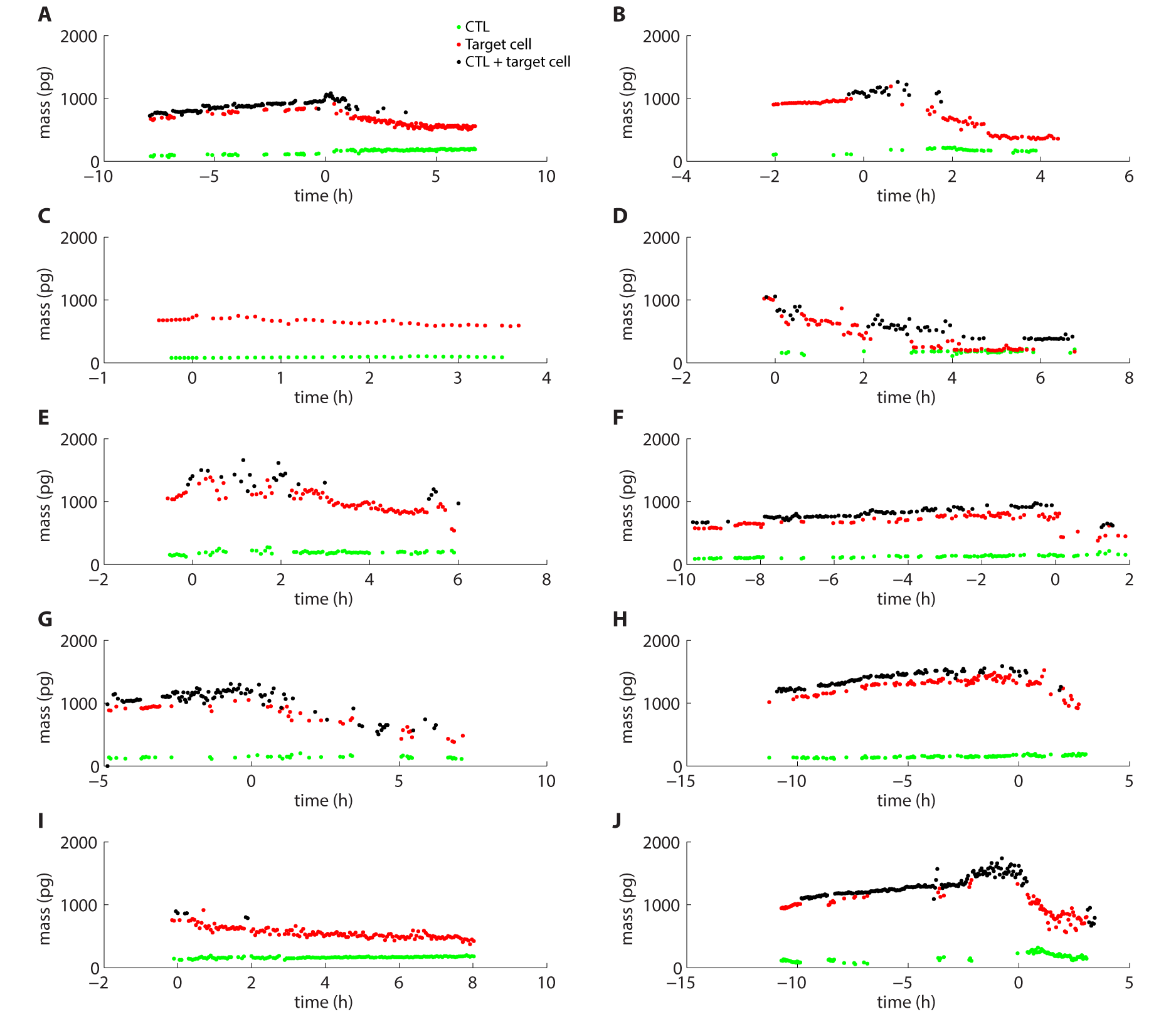

Supplement: Figure S4 — (A)–(J). Mass versus time plots for CTLs and corresponding target cells, as in Figure 4A . t = 0 h is the point at which the target cell detaches from the substrate at the beginning of cell death. CTL + target cell refers to total mass of both cells in frames where they could not be measured individually, typically due to overlap between the CTL and target cell. (TIF) [file pone.0068916.s004.tif]

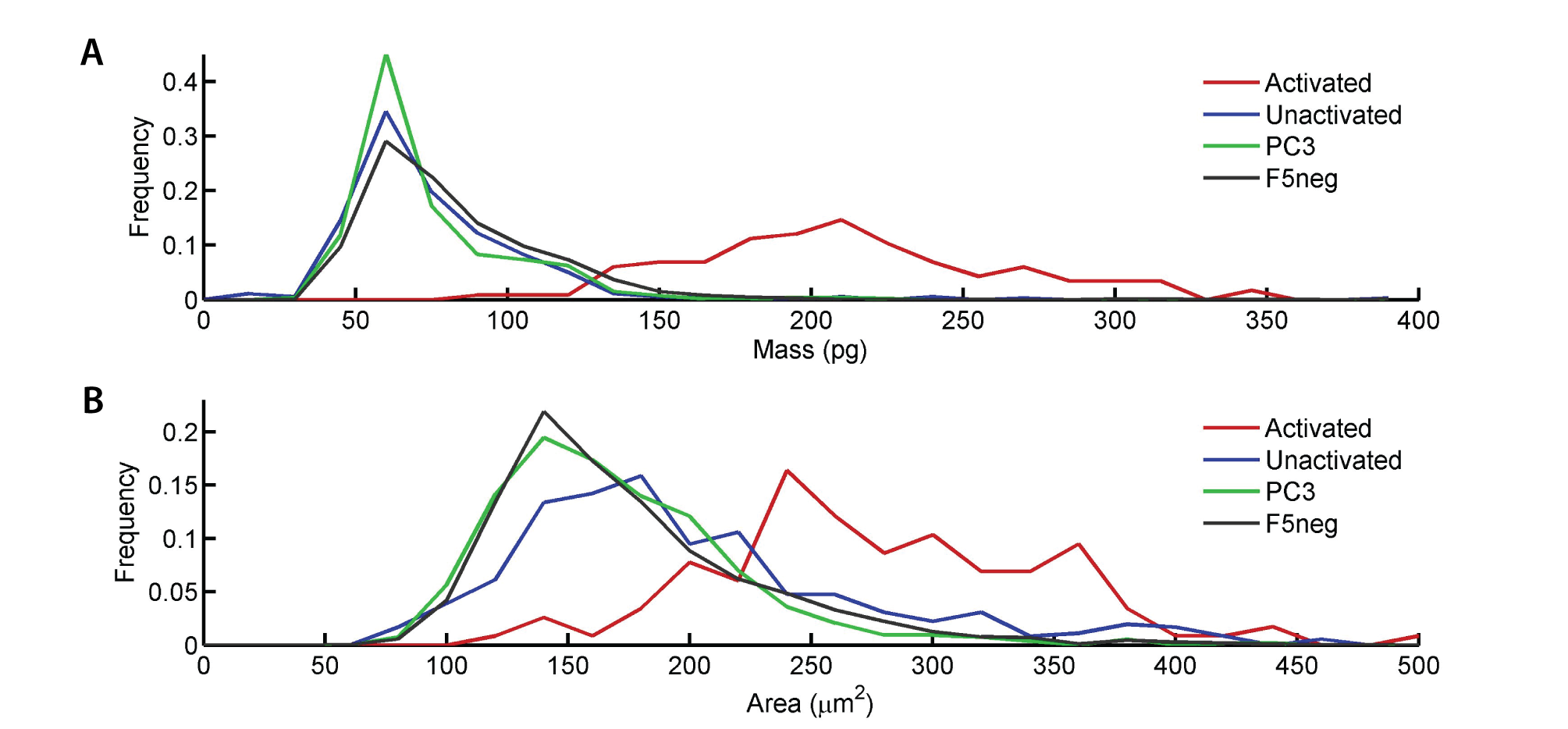

Supplement: Figure S5 — (A) Mass and (B) area histograms for activated and unresponsive T cells, relative to control experiments. Activated = activated/cytotoxic F5 TCR transduced T cells, 116 cells, n = 3 experiments. Unactivated = unactivated/unresponsive F5 TCR transduced T cells, 359 cells, n = 3 experiments. F5neg = untransduced F5 TCR negative T cells plated with M202 target cells, 530 T cells, n = 2 experiments. PC3 = F5 TCR transduced T cells plated with HLA-mismatched antigen irrelevant PC-3 prostate cancer cells, 3015 T cells, n = 3 experiments. (TIF) [file pone.0068916.s005.tif]
